# Genetic Diversity and Population Structure of *Rhododendron rex* Subsp. *rex* Inferred from Microsatellite Markers and Chloroplast DNA Sequences

**DOI:** 10.3390/plants9030338

**Published:** 2020-03-07

**Authors:** Xue Zhang, Yuan-Huan Liu, Yue-Hua Wang, Shi-Kang Shen

**Affiliations:** 1 School of Life Sciences, Yunnan University, Kunming 650091, China; 2 School of Ecology and Environmental Sciences & Yunnan Key Laboratory for Plateau Mountain Ecology and Restoration of Degraded Environments, Yunnan University, Kunming 650091, China; 3 Yunnan Key Laboratory of Plant Reproductive Adaptation and Evolutionary Ecology, Yunnan University, Kunming 650091, China; 4 Breeding Base for State Key Laboratory of Land Degradation and Ecological Restoration in Northwest China, Key Laboratory for Restoration and Reconstruction of Degraded Ecosystem in Northwest China of Ministry of Education, Ningxia University, Yinchuan 750021, China

**Keywords:** *Rhododendron*, conservation strategies, genetic differentiation, gene flow, populations contraction

## Abstract

Genetic diversity is vital to the sustainable utilization and conservation of plant species. *Rhododendron rex* subsp. *rex* Lévl. is an endangered species endemic to the southwest of China. Although the natural populations of this species are facing continuous decline due to the high frequency of anthropogenic disturbance, the genetic information of *R. rex* subsp. *rex* is not yet elucidated. In the present study, 10 pairs of microsatellite markers (nSSRs) and three pairs of chloroplast DNA (cpDNAs) were used in the elucidation of the genetic diversity, population structure, and demographic history of 11 *R. rex* subsp. *rex* populations. A total of 236 alleles and 12 haplotypes were found. A moderate genetic diversity within populations (*H_E_* = 0.540 for nSSRs, *Hd* = 0.788 for cpDNA markers), high historical and low contemporary gene flows, and moderate genetic differentiation (nSSR: *F_ST_* = 0.165***; cpDNA: *F_ST_* = 0.841***) were detected among the *R. rex* subsp. *rex* populations. Genetic and geographic distances showed significant correlation (*p* < 0.05) determined by the Mantel test. The species exhibited a conspicuous phylogeographical structure among the populations. Using the Bayesian skyline plot and species distribution models, we found that *R. rex* subsp. *rex* underwent a population demography contraction approximately 50,000–100,000 years ago. However, the species did not experience a recent population expansion event. Thus, habitat loss and destruction, which result in a population decline and species inbreeding depression, should be considered in the management and conservation of *R. rex* subsp. *rex*.

## 1. Introduction

*Rhododendron* is the largest woody plant genus in Ericaceae, containing more than 1000 recognized species, of which 567 species representing six subgenera are known from China [1]. Wild *Rhododendron* species are the major components of alpine and subalpine vegetation and widely distributed in America, Europe, and Asia, which have tropical to polar climates [2,3]. Therefore, these species are potential genetic resources for the development of new cultivars that can adapt to diverse environmental conditions [4]. In addition, plants in the genus *Rhododendron* L. produce numerous chemical constituents and are recognized as an important source of bioactive phytochemicals [5]. Some *Rhododendron* species are used as traditional medicine in China, India, Europe, and North America against various diseases, such as inflammation, pain, skin ailments, common cold, and gastrointestinal disorders [5]. However, as an important natural resource for human daily life and ecosystem composition, most *Rhododendron* species are facing risk of extinction due to the high frequency of anthropogenic disturbance [6]. Thus, research on the population genetic information of *Rhododendron* species is undoubtedly beneficial for germplasm protection and sustainable utilization [6,7,8,9].

Inferring genetic information is recognized as the undisputed basis for the sustainable exploitation and conservation of plant diversity [10,11]. Different molecular markers are used in assessing genetic information and identifying distinct plant populations for management and conservation [12,13,14]. Microsatellite markers (SSRs) are used in revealing the genetic characteristics and related influence factors of plant species at individual and population levels due to their desirable advantages [13,15]. Chloroplast DNA (cpDNA), which is transmitted only through seeds in most angiosperms, is exceptionally conserved in gene content and organization, providing sufficient information for genome-wide evolutionary studies [16]. cpDNA can reveal a more highly geographical structure than a nuclear genome [17] and is generally used in the detection of phylogeographical patterns in plant species [18,19]. Thus, nSSRs and cpDNA were extensively and successfully documented to study the genetic diversity, variation, and population demographic of plant species [17,20,21,22].

Habitat loss and destruction are global problems that continue to threaten global biodiversity [23,24]. Firstly, habitat destruction and loss can cause a decline in the distribution range and population and limit the natural regeneration of a species. Secondly, habitat destruction and loss can increase selfing rates and decrease pollen diversity, thereby affecting a species’s reproductive success [23,25]. Finally, habitat destruction and loss increase genetic drift and inbreeding and reduce gene flow in the fragmented populations of species and substantially decrease species genetic diversity and adaptation to the changing environment. Some studies suggested that woody plants are less likely to lose genetic diversity after habitat fragmentation and destruction than herbaceous species [26]; however, recent reports showed that habitat loss and fragmentation are associated with increased level of inbreeding, reduced gene flow, genetic variation, plant progeny quality, and genetic extinction debt in woody species [24,27]. Thus, understanding the current genetic information of endangered woody plants subjected to habitat loss and destruction is necessary for effective conservation and management.

*Rhododendron* species are not only popular woody ornamental plants but also play an important role in alpine and subalpine ecosystems. In addition, *R. rex* is an important wild germplasm source of the genus *Rhodendron* in China and an endangered plant endemic to the southwest of China [1]. Three subspecies (*R. rex* subsp. *rex*, *R. rex* subsp. *gratum*, and *R. rex* subsp. *fictolacteum*) are recognized in the *R. rex* complex. Recently, the wild populations of *R. rex* subsp. *rex* are facing continuous decline due to the high frequency of anthropogenic disturbance and forest destruction. Genetic information is important to the management and sustainable exploitation of species, particularly those threatened by habitat loss and destruction. However, the genetic diversity and structure of the wild populations of *R. rex* subsp. *rex* remain unexplored. In the present study, the genetic diversity and differentiation, population structure, and demographic history of 11 *R. rex* subsp. *rex* populations are inferred using 14 pairs of microsatellite markers and three cpDNA sequences. The following central questions are addressed: (1) What is the level of genetic diversity in *R. rex* subsp. *rex*? How does they apportion among/within the populations? (2) How is the genetic structure of the remnant population? Are they affected by historical and contemporary gene flows? (3) How is the phylogenetic relationship of haplotypes? Are they reflected by the demographic history in *R. rex* subsp. *rex*? This result is used to design optimum management strategies for *R. rex* subsp. *rex* conservation.

## 2. Materials and Methods

### 2.1. Plant Material Sampling

**Figure 1:**
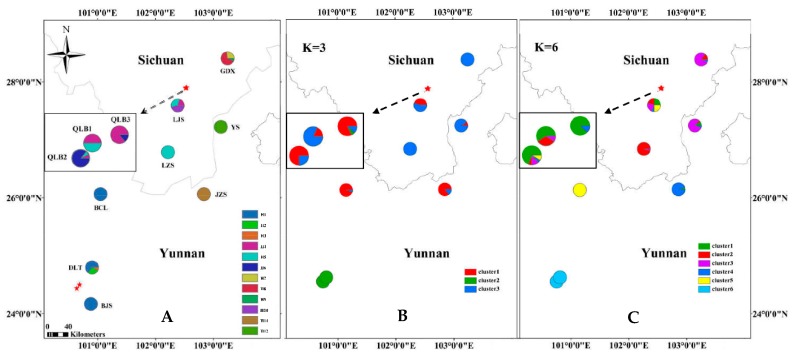
Distribution of chloroplast DNA (cpDNA) haplotypes (**A**); map of the geographic distribution of nuclear microsatellite clusters when the assumed cluster numbers are (**B**) *K* = 3 and (**C**) *K* = 6 in 11 populations of *Rhododendron rex subsp. rex*.

We collected 212 individuals of *R. rex* subsp. *rex* from 11 natural populations. Four of these populations (BJS, DLT, BCL, and JZS) with 63 individuals were distributed in Yunnan province, whereas seven populations (QLB1, QLB2, QLB3, GDX, LJS, LZS, and YS) with 149 individuals were distributed in Sichuan province, China (Table 1). Our sampling locations covered all the herbarium sampling sites and documented sites of *R. rex* subsp*. rex*. During field sampling, sampled site, sampled individuals, and altitude were recorded (Figure 1 and Table 1). Fresh leaves were collected from individuals of *R. rex* subsp*. rex* separated by a minimum distance of 15 m and then dried in silica gel immediately. The samplings were stored at −4 °C until DNA extraction.

### 2.2. DNA Extraction, PCR Amplification, and Sequencing

We extracted genomic DNA of *R. rex* subsp*. rex* from the silica-dried leaves through a modified cetyltrimethyl ammonium bromide (CTAB) method [28]. Purified DNA was amplified by three universal cpDNA sequences (*rbc*L, *mat*K, and *psb*A*-trn*H). A total of 14 SSR markers were selected from recently developed nuclear microsatellites in *Rhododendron* subg. *Hymenanthes* according to their clarity and reproducibility (Table S1) [29,30,31]. PCR amplification was performed in accordance with methods of Zhang et al. [1]. Forward SSR primers were labeled with a fluorescent dye (FAM, TAMRA, or HEX) and visualized by an ABI 3730xl automated sequencer at Sangon Biotech Services Company Ltd. (Shanghai, China). Fragment sizes were read with the GeneMapper version 4.0. CERVUS [32] was used in eliminating four loci as existing null alleles (*F*_(Null)_ > 0.4) [33]. PCR products by three cpDNA intergenic spacers were sequenced in both directions by Sangon Biotech Services Company Ltd. (Shanghai, China).

### 2.3. Data Analysis

#### 2.3.1. Data Analysis of Microsatellite Markers

The dataset was edited and formatted with GenAlEx ver. 6.3 [34]. We used Genepop ver. 4.1.4 to test the Hardy–Weinberg equilibrium (HWE) for each locus and population [35]. The universal genetic diversity parameters were calculated using GenAlEx ver. 6.3 [34] and POPGENE ver. 1.32 [35]. Then, rarefied allelic richness (*Ra*), total diversity (*H_T_*), and the level of gene differentiation (*G_ST_*) among *R. rex* subsp. *rex* populations were estimated by FSTAT ver. 2.9.3 [13,36]. Analysis of the molecular variance (AMOVA) was implemented in the estimation of genetic variation by using Arlequin ver. 3.11 [37,38], and *F_ST_* values with 10^3^ permutations were calculated for the assessment of genetic differentiation between the pairwise populations of *R. rex* subsp*. rex.*

The historical gene flow (*Nm*) between the pairs of *R. rex* subsp*. rex* populations was calculated using Wright’s principles using formula *Nm* = (1 − *F_ST_*)/4*F_ST_* [39]. In addition, pollen to seed gene flow ratio (m_p_/m_s_) was calculated using the Ennos formula [40]. To estimate contemporary migration patterns, we estimated the contemporary inter-population migrations in *R. rex* subsp. *rex* using the BayesAss version 3.0 by 3 × 10^6^ Markov chain Monte Carlo (MCMC) iterations, with a burn-in of 10^6^ iterations and a sampling frequency of 2000 by setting delta at 0.15 (the default value) [41,42,43].

Isolation by distance was examined in GenAlEx ver. 6.3 on the basis of the correlation of a geographic distance for pairwise populations with *F_ST_*/(1 − *F_ST_*) value [34]. Population structure was accessed through unweighted pair group mean analysis (UPGMA) and principal coordinate analysis. TFPGA ver. 1.3 with 5000 permutations [44] and GenAlEx ver. 6.3 [34] were used, respectively. The Bayesian clustering analysis with an admixture model to understand the population structure of *R. rex* subsp*. rex* using STRUCTURE ver. 2.2 was also explored [22,45]. *K*-values in the model ranged from two to 15 with 20 independent variables for each set with a burn-in of 1 × 10^5^ iterations and 1 × 10^5^ subsequent Markov chain Monte Carlo steps [45]. The final best-fit number of the clusters was determined by Δ*K* values in STRUCTURE HARVESTER ver. 0.6.8 [46,47].

By performing a heterozygosity excess test, we explored the demographic history of the populations. We used two different models, namely, stepwise mutation and two-phased models, to construct the recent bottleneck statistic in BOTTLENECK ver. 1.2.02 (Sign and Wilcoxon tests) [48]. We further analyzed the genetic bottleneck with Garza–Williamson index (GWI) in Arlequin ver. 3.11 [38]. GWI lower than the critical *Mc* value of 0.68 indicated a reduction in population size [1,38,49].

#### 2.3.2. Data Analysis of cpDNA Sequences

We used SeqMan II [50] and Bioedit ver. 7.0.4.1 [51] to treat the raw sequencing data and manually edited and assembled these sequences [22]. Three cpDNA intergenic spacers of *R. rex* subsp. *rex* were combined using PAUP 4.0 [52].

The haplotypes and variable sites of combined cpDNA sequences were calculated by DnaSP ver. 5.0 [53]. *Nei*’s nucleotide diversity (*Pi*) and haplotype diversity (*Hd*) indices of *R. rex* subsp. *rex* were tested within a population and among populations. The haplotype distribution in each sampled population was plotted by ArcGIS ver. 10.2. In addition, we calculated *H_T_* and within-population gene diversity (*H_S_*) with Permut ver. 1.0 [22]. The two values of population differentiation *G_ST_* and *N_ST_* were computed in accordance with the methods described by Pons and Petit [54] and with the same software. AMOVA of cpDNA sequences was constructed with Arlequin ver. 3.11 [37,38].

A genealogical haplotype network was constructed by Network ver. 4.2.0.1 for the estimation of the relationship per haplotype, and an indel was treated as a single mutational event [55]. The phylogenetic relationships of the as-obtained haplotypes of *R. rex* subsp. *rex* were inferred by Bayesian methods and neighbor-joining method in MrBayes ver. 3.1.2 [56]. *Nerium oleander* (EU916729.1, GQ997664.1 and AY899942.1) was selected as the outgroup species.

The evolutionary rates of seed plants (1.01 × 10^−9^) were used for each Beast analysis in BEAST ver. 1.6.1 [57,58,59] with 10^7^ iterations and a burn-in of 10^6^ under the Hasegawa–Kishino–Yano (HKY) model and a strict clock. The most suitable model (HKY) was determined by Mega ver. 6.06 [60]. The results were visualized using the software FigTree ver. 1.4.2. The signatures of demographic changes in *R. rex* subsp. *rex* populations were assessed. We calculated pairwise mismatch distribution, neutrality tests (Tajima’s D and Fu’s F_S_), the sum of squared deviations and the raggedness index, and their *p*-values using DnaSP ver. 5.0 [53] and Arlequin ver. 3.11 [38].

#### 2.3.3. Analysis of Species Distribution Model

Species distribution models were constructed for the identification of the species’ potential distribution during the last glacial maximum (LGM; ~21–18 ka) at present and in the future (model rcp45 for the years 2050, model rcp85 for the years 2070) by MAXENT v. 3.3.3k [61]. For each time period, models were run for 25 replicates, and default parameters were used. A total of 28 points comprised our 11 sampled sites and 17 records compiled in the Chinese Virtual Herbarium (www.cvh.org.cn), and 19 bioclimatic variables were obtained from the WorldClim database [62].

## 3. Results

### 3.1. SSR Data

We identified 169 alleles at 10 polymorphic loci among 11 *R. rex* subsp. *rex* populations, ranging from eight (R-40, R-49) to 30 (R-30), with an average of 16.9 alleles per locus (). All loci and populations conformed to HWE (*p* > 0.05; Table S3). At the locus level, genetic diversity and variation exhibited certain dissimilarities. However, no remarkable difference was detected between populations (Table 2). *N_P_* varied from 2 (BJS) to 12 (DLT and JZS), *Ra* varied from 3.071 (BJS) to 4.231 (JZS), *A_E_* varied from 2.011 (BJS) to 3.954 (YS), and *I* varied from 0.740 (BJS) to 1.319 (YS). The minimum values of *H_O_* (0.300) and *H_E_* (0.399) occurred in population BJS. The mean value of fixation indices (*Fis* = 0.171; Table 2) was positive for *R. rex* subsp. *rex* populations, suggesting a slightly increased level of inbreeding.

AMOVA indicated that 83.53% genetic variation occurred within populations, whereas 16.47% variation was estimated among the populations (Table 3). Genetic differentiation was observed among populations (*F_ST_* = 0.165, 0.15 < *F_ST_* < 0.25).

A high level of historical gene flow of pairwise populations was detected in *R. rex* subsp. *rex* (Table 4). The minimum gene flow was generated from populations BJS and QLB2 (0.307), whereas the maximum gene flow was generated from populations QLB2 and QLB3 (7.452). The relative contribution of m_p_/m_s_ was 24.775, indicating that pollen dispersal played an important role in the gene flow of *R. rex* subsp. *rex*. Except for other populations migrating to LJS, a non-significant level of inter-population contemporary migration rate between the populations of *R. rex* subsp. *rex* was detected (*M* < 0.05, Table 5).

**Figure 2:**
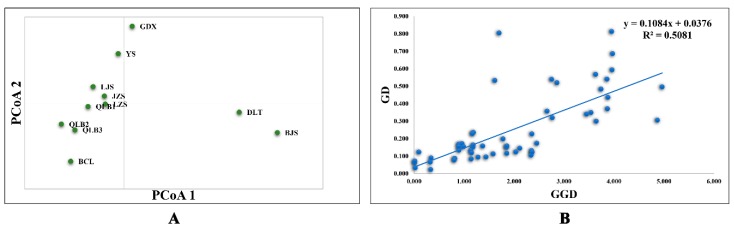
Principal coordinate analysis (**A**) and the plot of geographical distance against genetic distance (**B**) for *R. rex* subsp. *rex* by SSR data analysis.

The optimal *K* value was 3 with Δ*K* of 63.924, and the second fit value was 6 with Δ*K* of 16.473 according to STUCTURE analysis (Figure S1A,B). At *K* of 3, the populations GDX, YS, and JZS were similar, BJS and DLT were related, and the remaining populations BCL, LJS, LZS, QLB1, QLB2, and QLB3 comprised one group (Figure 1B; Figure S1A). At *K* = 6, the populations BCL and LZS were further distinguished from LJS, QLB1, QLB2, and QLB3, and JZS was further distinguished from GDX and YS (Figure 1C; Figure S1A). This result was in accordance with the conclusions of UPGMA (Figure S1C) and principal component analysis (PCA) at the population level (Figure 2A). In conclusion, the populations of *R. rex* subsp. *rex* should be grouped into three groups according to the genetic structure analysis results by using SSR data. A significant correlation between genetic and geographic distances was determined by Mantel test (*p* < 0.050; Figure 2A).

As shown in Table 6, most of the probabilities of the Wilcoxon and Sign tests under both models in *R. rex* subsp. *rex* populations were non-significant (*p* > 0.05). In addition, the allele distribution per population was presented as a normal L-shaped distribution. The above-mentioned results indicated that the *R. rex* subsp. *rex* populations conformed to the mutation–drift equilibrium. However, GWI values were lower than the critical *M*c indices (0.68), implying that the populations of *R. rex* subsp. *rex* underwent a demographic reduction in history.

### 3.2. cpDNA Sequence

The three cpDNAs, *mat*K, *psb*A-*trn*H, and *rbc*L were 828, 398, and 658 bp in length, respectively (GenBank accession numbers: MN228019-MN228483). The 1884-bp combined cpDNA sequences of *R. rex* subsp. *rex* had 18 polymorphic sites and 12 haplotypes (H1–H12) (Table 1). The detailed haplotype distribution per population is displayed in Figure 1.

The populations DLT (0.538 and 0.00031) and GDX (0.514 and 0.00115) exhibited additional maximum values of *Hd* and *Pi* per site, followed by QLB2 (*Hd* = 0.264, *Pi* = 0.00028) and QLB3 (*Hd* = 0.264, *Pi* = 0.00028), whereas no diversity was found in the seven remaining populations (Table 1). In summary, the total *Hd* and *Pi* for *R. rex* subsp. *rex* were 0.78768 and 0.00180, respectively. The value of *H_T_* (0.909) was higher than that of *H_S_* (0.337), and the value of *N_ST_* (0. 0.774) was significantly higher than that of *G_ST_* (0.629; *p* < 0.05; Table S4). These results indicated the remarkable phylogeographic structure among the populations of *R. rex* subsp. *rex*. AMOVA indicated that 84.07% genetic variation was partitioned among populations, whereas 15.93% was partitioned within populations (Table 3). This result was inconsistent with the result of nSSRs data. Moreover, significant genetic differentiation was observed among *R. rex* subsp. *rex* populations (*F_ST_* = 0.841, *p* < 0.001).

**Figure 3:**
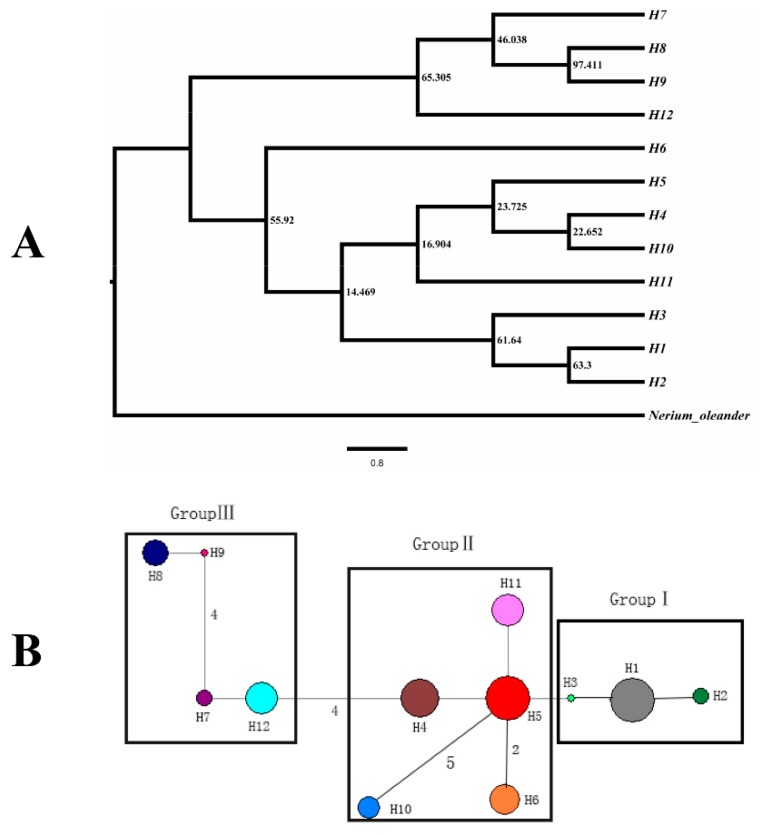
Bayesian tree (**A**) and the network of haplotypes (**B**) based on combined cpDNA sequences. (**A**) The numbers on branches indicate the posterior probability; (**B**) the size of the circles corresponds to the frequency of each haplotype, and the vertical branches indicate mutational steps.

The phylogenetic relationships of 12 cpDNA haplotypes are shown in Figure 3A. H7, H8, H9, and H12 were grouped into one clade. Among the remaining haplotypes, H1, H2, and H3 were grouped into one clade, while H4, H5, H10, and H11 were grouped into another clade. H6 was differentiated from the remaining others. However, the result of the haplotype network diagram shown that H6 distributed more closely to H5, and the 12 cpDNA haplotypes should be divided into three groups (Figure 3B).

**Figure 4:**
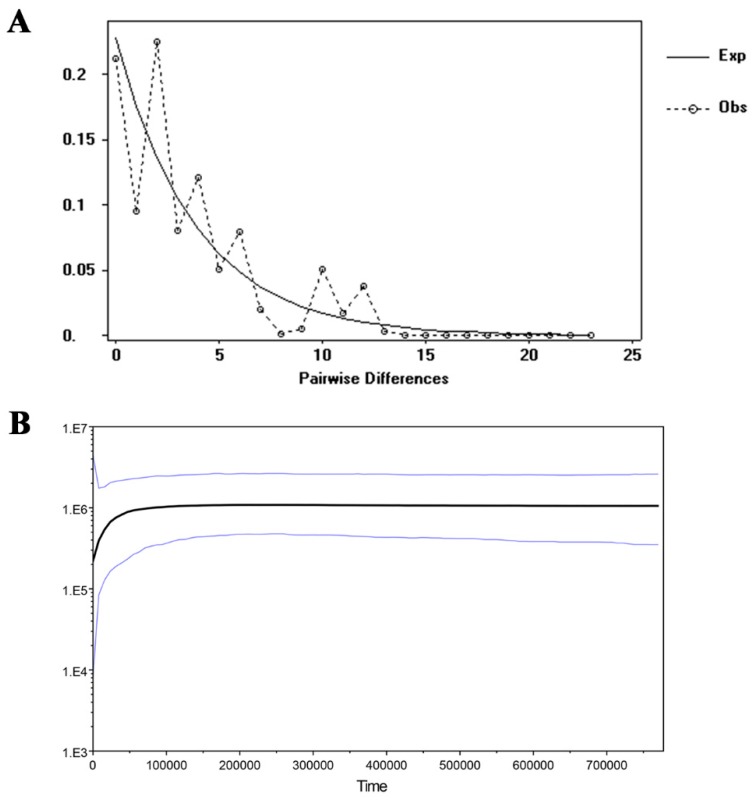
Mismatch distribution (**A**) and Bayesian skyline plot based on combined cpDNA sequences (**B**). (**A**) The solid lines show expected values, whereas the dashed lines represent observed values under a model of sudden population expansion. (**B**) The black line indicates effective population size fluctuation throughout.

Only the Fu and Li’*D* yielded significantly positive values (*p* < 0.05; Table S5) according to the neutrality test. This result indicated that no recent population expansion in *R. rex* subsp. *rex* occurred, and this was supported by the effects of mismatch distributions shown in the multimodal graph (Figure 4A). Based on the Bayesian analysis, the skyline plot indicated that the historical demographic of *R. rex* subsp. *rex* populations experienced a contraction event approximately 50,000–100,000 years ago and had no recent expansion (Figure 4B).

### 3.3. Species Distribution Model

**Figure 5:**
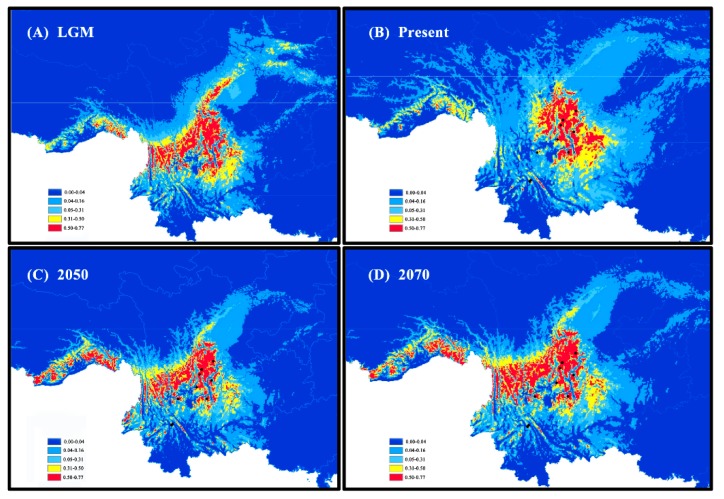
Distribution dynamics of *R. rex* subsp. *rex* using MAXENT. Predicted distributions are shown for (**A**) the last glacial maximum (LGM), (**B**) the present, (**C**) the year 2050, and (**D**) the year 2070. Color-coded keys represent different habitat suitability.

According to predictions of *R. rex* subsp. *rex*’s past, present, and future potential distributions, the predicted current distributions showed a clear range contraction relative to the LGM distributions. Moreover, the moderate habitat suitability (>0.31) was slightly removed to the northeastern direction (Figure 5A,B). The potential distribution with moderate to high habitat suitability (>0.31) for the years 2050 and 2070 was slightly extended compared with the present-day model (Figure 5C,D).

## 4. Discussion

### 4.1. Genetic Diversity in R. rex Subsp. rex Populations

The genetic diversity of species reflects its long-term evolution and adaptation demographic history [63]. Based on nSSR data, *R. rex* subsp. *rex* has lower genetic diversity (*H_E_* = 0.540) than the other species of *Rhododendron*, such as *R. protistum* var. *giganteum* (*H_E_* = 0.602) [9], *R. jinggangshanicum* (*H_E_* = 0.642) [64], *R. simsii* (*H_E_* = 0.754) [65], *R. ripense* (*H_E_* = 0.800) [66], and *R.* *brachycarpum* (*H_E_* = 0.815) [67], but has higher genetic diversity than *R. ferrugineum* (*H_E_* = 0.500) [68]. Meanwhile, the genetic diversity of *R. rex* subsp. *rex* is evidently higher than that of the “narrow” species (*H_E_* = 0.420) and lower than that of the “widespread” species (*H_E_* = 0.620) [69]. Inconsistent with the results of microsatellite markers, the genetic diversity of *R. rex* subsp. *rex* (*Pi* = 0.00180) assessed by cpDNA shows a higher tendency toward high genetic diversity than the genetic diversities of 20 species of *Rhododendron* sect. *Brachycalyx* (insular species, *Pi* = 0.00040; continental species, *Pi* = 0.00160) in East Asia [70], but a lower tendency than the genetic diversity of bird-dispersed arctic–alpine plant *Vaccinium vitis-idaea* in Ericaceae (*Pi* = 0.00240) [71]. The value of *H_T_* estimated in *R. rex* subsp. *rex* (0.909) is higher than the mean value of *H_T_* (0.6747) in 170 plant species according to cpDNA [19,72]. Therefore, *R. rex* subsp. *rex* possesses a relatively moderate genetic diversity compared with the other species in *Rhododendron*. In general, life span, reproductive mode, and breeding system are the important factors in genetic diversity [6,22,69]. As in other outcrossing and long-lived species in *Rhododendron*, high historical gene flow among ancestral population mitigates the loss of genetic diversity and further results in a moderate or high genetic diversity in remnant populations [69,73]. Thus, the current levels of genetic diversity in *R. rex* subsp. *rex* may be attributed to the species’ long-lived habit, which is similar to other perennial woody plants [9,22].

### 4.2. Genetic Differentiation and Structure among R. rex Subsp. rex Populations

The *F_ST_* value of *R. rex* subsp. *rex* indicated that a moderate genetic differentiation among populations occurred. A total of 83.75% genetic variation occurred within *R. rex* subsp. *rex* populations with regard to nSSR markers, whereas 83.53% variation was partitioned among populations with regard to cpDNA sequences. This discordance should be affected by dispersal mechanisms among populations of plant species in *Rhododendron* [74,75]. Insect visitors are the primary pollen dispersal vectors for *Rhododendron* species. Various insect vectors evolved longer dispersal distance for pollen, whereas the seeds dispersed by wind traveled less than 10 m albeit in open landscapes [74]. Meanwhile, this different consequence might be related to the type and evolutionary rates of different genome sequences [76]. In general, the evolutionary rate of nuclear genomes transmitted by parents was higher than that of maternally inherited chloroplast genomes [77]. Therefore, cpDNA variations reflected a past change, whereas nSSR variations inferred recent events in the population demographics of *R. rex* subsp. *rex*.

On the basis of genetic structure analysis by SSR data, the populations of *R. rex* subsp. *rex* were grouped into three groups, and the correlation between genetic and geographic distances was significant (*p* < 0.05). Phylogenetic trees and genealogical haplotype networks based on cpDNA sequences showed that three reciprocally lineages were detected. This species possessed unique genetic lineages and endemic cpDNA haplotypes in its separate refuge populations. The closely related haplotypes H1, H2, and H3 were distributed in populations BJS, BCL, and DLT; H4, H5, H6, H10, and H11 occurred primarily in populations LJS, JZS, LZS, QLB1, QLB2, and QLB3; H7, H8, H9, and H12 were only detected in populations GDX and YS.

Habitat dislocation and overexploitation accelerate the generation of genetic differentiation among populations [9,13]. In the sampled regions, large-scale land reclamation and unreasonable forest destruction can be observed, which resulted in habitat loss and fragmented distribution in *R. rex* subsp. *rex* natural populations. In addition, gene flow is a fundamental micro-evolutionary force, influencing genetic differentiation among populations [78,79]. The contemporary gene flow of *R. rex* subsp. *rex* is lower than that of the related species of *R. protistum* var. *giganteum* [9], which plays an important role in the formation of genetic structure and differentiation among *R. rex* subsp. *rex* populations. Moreover, breeding system is an important factor for the genetic differentiation and structure of a species [6,22,69]. Although both selfing and outcrossing are detected in *Rhododendron* species [80,81], the breeding system in *R. rex* subsp. *rex* is yet to be explored. Based on the positive value of fixation indices (*F_is_*) and the phenomenon of all populations deviated from HWE in the present study, we can reasonably speculate that inbreeding is present in populations of *R. rex* subsp. *rex*. Hence, the mating system and its influences on genetic differentiation and structure must be further elucidated in *R. rex* subsp. *rex*.

### 4.3. Population Demographic History of the R. rex Subsp. rex

Exploring the historical demography of a species can facilitate our knowledge of its ancient evolutionary environment [58]. Quaternary glaciers profoundly affected the distribution and genetic variation of plant species. Tremendous global climatic oscillations during quaternary glaciations with several glacial–interglacial cycles caused the expansion and contraction of plant distribution [82]. Most plants were subjected to population demographic stability or expansion throughout the LGM [83,84]. The Bayesian skyline plot of cpDNA showed that *R. rex* subsp. *rex* experienced a notable reduction approximately 50,000–100,000 years ago. This supposition is supported by the GWI values, which are lower than the critical *M*c indices (0.68). Microsatellite-based bottleneck analysis indicated that no recent bottleneck event occurred in the natural populations of *R. rex* subsp. *rex*. Therefore, the population demographic contraction detected in the *R. rex* subsp. *rex* might have been the result of climate oscillations, and the finding is consistent with the results obtained from other species, such as *Cycas simplicipinna* [63]. Typically, rapid population expansion occurred in the post-glacial period because temperatures increased to warm conditions [85]. However, based on neutrality and mismatch distribution tests, no recent population expansion occurred in *R. rex* subsp. *rex*. We speculate that the populations of *R. rex* subsp. *rex* might have survived in situ rather than migrating long distances to suitable habitats and that evolutionary adaptation might have occurred in the cold environment. The current existing populations of *R. rex* subsp. *rex* were limited in distribution at 2400–3400 m elevation, and this condition might partly support our speculation. In addition, the complex topology of physical environmental condition in southwest China might cause geographical barriers between population migrations. This scenario was also found in the population demography of *Leucomeris decora* [86].

## 5. Conclusions

The present study firstly investigated the genetic diversity, population structure, and demographic history of 11 remnant *R. rex* subsp. *rex* populations. A moderate genetic diversity, a high genetic differentiation, and a conspicuous geographical structure were detected in *R. rex* subsp. *rex*. The species possessed unique genetic lineages and endemic cpDNA haplotypes in its separate refuge populations. In addition, we found that *R. rex* subsp. *rex* experienced a population contraction approximately 50,000–100,000 years ago based on the comprehensive analysis of demographic history. Furthermore, no recent population expansion occurred in this species. Hence, the conservation of *R. rex* subsp. *rex* should focus on habitat destruction and loss, resulting in a population decline and inbreeding depression within populations. Furthermore, all the remnant adult trees of *R. rex* subsp. *rex* should receive priority protection for the maintenance of its genetic diversity. This research exhibited tremendous ecological value for the future conservation and sustainable utilization of *R. rex* subsp. *rex* and other similar plants, which are subjected to climate oscillation, inbreeding depression, overexploration, and habitat destruction.

## Figures and Tables

**Table 1:** Details of sample locations, sample size (*N*), haplotype diversity (*Hd*), and nucleotide diversity (Pi) surveyed for cpDNA sequences of *R. rex* subsp. *rex.* SSR—microsatellite marker.

Location	Population Code	Latitude	Longitude	Altitude (m)	*N* (cpDNA/SSR)	Haplotypes (No.)	cpDNA	
							*Hd*	*Pi*
Yunnan	BJS	24°24′31″	100°38’15″	2670	**6/6**	H1	0	0
	DLT	24°28′57″	100°41’47″	2660	14/15	H1, H2, H3	0.538	0.00031
	BCL	26°3′26″	101°03’11″	2950	15/21	H1	0	0
	YS	27°13′09″	103°07’43″	2887	16/23	H12	0	0
	JZS	26°04′07″	102°49’56″	3250	16/21	H11	0	0
Sichuan	QLB1	27°53′46″	102°30’56″	3250	14/22	H4, H5	0	0
	QLB2	27°53′19″	102°30’36″	3303	14/23	H4, H5, H6	0.264	0.00028
	QLB3	27°54′0.4″	102°31’44″	3332	14/17	H4, H6	0.264	0.00028
	GDX	28°24′29″	103°14’33″	2966	15/20	H7, H8, H9	0.514	0.00115
	LJS	27°35′19″	102°23’34″	2833	15/20	H4, H5, H10	0	0
	LZS	26°47′48″	102°12’30″	3335	16/24	H5	0	0
Total	11				155/212	H1–H12	0.788	0.0018

**Table 2:** Genetic diversity of populations in *R. rex* subsp. *rex.*

Population	*N_P_*	*Ra*	*N_A_*	*A_E_*	*I*	*H_O_*	*H_E_*	*Fis*	*PPB* (%)
BCL	10	3.574	5.800	3.215	1.061	0.429	0.474	0.119	100.00%
BJS	2	3.071	3.100	2.011	0.740	0.300	0.399	0.331	90.00%
DLT	12	4.178	6.100	3.804	1.281	0.513	0.578	0.148	90.00%
GDX	3	3.681	5.800	3.479	1.183	0.452	0.547	0.200	100.00%
JZS	12	4.231	6.100	3.085	1.252	0.401	0.605	0.357	100.00%
LJS	8	3.841	6.700	3.169	1.228	0.478	0.561	0.167	100.00%
LZS	7	3.676	6.200	3.114	1.230	0.558	0.585	0.068	90.00%
QLB1	4	3.689	5.800	3.213	1.183	0.515	0.556	0.098	100.00%
QLB2	3	3.618	6.200	2.994	1.086	0.417	0.486	0.165	100.00%
QLB3	5	3.718	5.900	3.118	1.187	0.498	0.541	0.111	90.00%
YS	11	3.937	6.900	3.954	1.319	0.547	0.605	0.119	100.00%
Mean	7	3.747	5.873	3.196	1.159	0.464	0.540	0.171	96.36%

Note: *N_A_*, mean number of alleles; *A_E_*, number of effective alleles; *I*, Shannon’s information index; *H_O_*, observed heterozygosity; *H_E_*, expected heterozygosity; *N_P_*, number of private alleles; *Ra*: rarefied allelic richness; *Fis*, fixation index; *PPB* (%), percentage of polymorphic loci.

**Table 3:** Analysis of molecular variance (AMOVA) based on 14 microsatellites and three cpDNA sequences in *R. rex* subsp. *rex.* d.f.: degrees of freedom.

	Source of Variation	d.f.	Sum of Squares	Variance Components	Percentage of Variation (%)	
SSR data	Among populations	10	237.748	0.548	16.47	*F*_ST_ = 0.165 ***
	Within populations	413	1148.398	2.781	83.53	
	Total	423	1386.146	3.329		
cpDNA sequences	Among populations	10	276.023	1.940	84.07	F*_ST_* = 0.841 ***
	Within populations	144	52.919	0.367	15.93	
	Total	154	328.942	2.314		

Note: *** *p* < 0.001, most significant difference.

**Table 4:** Historical gene flows between 11 populations of *R. rex* subsp. *rex.*

Population	BCL	BJS	DLT	GDX	JZS	LJS	LZS	QLB1	QLB2	QLB3	YS
BCL	0										
BJS	0.311	0									
DLT	0.469	2.024	0								
GDX	0.734	0.504	0.817	0							
JZS	1.257	0.463	0.699	1.439	0						
LJS	2.013	0.439	0.715	2.62	2.208	0					
LZS	1.582	0.481	0.781	1.721	1.454	2.86	0				
QLB1	1.895	0.421	0.674	1.636	2.146	10.591	2.97	0			
QLB2	2.363	0.307	0.462	1.059	1.649	3.782	1.882	4.021	0		
QLB3	2.029	0.365	0.572	1.093	1.582	2.785	2.099	3.461	7.452	0	
YS	1.100	0.517	0.833	2.642	1.525	3.207	1.621	1.862	1.479	1.617	0

**Table 5:** Contemporary migration rate between populations of *R. rex* subsp. *rex* by BayesAss with 95% confidence intervals.

Population->	BCL	BJS	DLT	GDX	JZS	LJS	LZS	QLB1	QLB2	QLB3	YS
BCL	0.695	0.029	0.029	0.028	0.027	0.055	0.028	0.028	0.027	0.028	0.028
BJS	0.029	0.696	0.028	0.028	0.028	0.051	0.028	0.028	0.029	0.027	0.029
DLT	0.029	0.027	0.695	0.028	0.028	0.053	0.027	0.027	0.029	0.028	0.029
GDX	0.028	0.027	0.027	0.697	0.029	0.052	0.027	0.028	0.028	0.028	0.028
JZS	0.027	0.028	0.028	0.026	0.695	0.057	0.027	0.028	0.028	0.029	0.027
LJS	0.030	0.028	0.027	0.029	0.028	0.719	0.027	0.028	0.029	0.027	0.029
LZS	0.028	0.028	0.029	0.028	0.028	0.057	0.694	0.027	0.026	0.028	0.027
QLB1	0.029	0.027	0.027	0.028	0.027	0.055	0.027	0.694	0.028	0.029	0.028
QLB2	0.029	0.028	0.026	0.029	0.027	0.055	0.028	0.029	0.695	0.027	0.027
QLB3	0.028	0.029	0.028	0.028	0.027	0.055	0.027	0.028	0.028	0.695	0.028
YS	0.028	0.028	0.026	0.028	0.027	0.056	0.028	0.026	0.029	0.028	0.695

Note: population->: population migration into the other populations.

**Table 6:** Bottleneck analysis of 11 populations in *R. rex* subsp. *rex.*

Population	Two Phased Model (T.P.M)		Step Mutation Model (S.M.M)		Mode Shift	Garza–Williamson Index
	Sign Test	Wilcoxon Test	Sign Test	Wilcoxon Test		
BCL	0.614	0.539	0.170	0.813	L	0.336
BJS	0.211	0.410	0.068	0.545	L	0.399
DLT	0.399	0.652	0.183	0.839	L	0.329
GDX	0.158	0.862	0.002 **	0.998	L	0.492
JZS	0.176	0.813	0.183	0.958	L	0.361
LJS	0.178	0.862	0.169	0.958	L	0.278
LZS	0.074	0.947	0.003 **	0.995	L	0.297
QLB1	0.370	0.423	0.181	0.947	L	0.284
QLB2	0.371	0.461	0.058	0.984	L	0.323
QLB3	0.065	0.862	0.074	0.984	L	0.333
YS	0.612	0.461	0.389	0.722	L	0.349

Note: ** *p* < 0.01, significant difference.

## Supplementary Materials

The following are available online at https://www.mdpi.com/2223-7747/9/3/338/s1: Figure S1. Bayesian inference of the number of clusters when *K* = 3 and *K* = 8 (A), delta *K* values obtained (B), and an unweighted pair-group method with arithmetic averages (UPGMA) phenogram of *R. rex* subsp. *rex* (C) based on nSSR; Table S1. The information of 14 microsatellite primers for the *R. rex* subsp. *rex*; Table S2. Summary of the 10 microsatellite loci used to the 11 populations of *R**. rex* subsp. *rex**;* Table S3. *p*-Value of Hardy–Weinberg equilibrium test for 11 populations of *R. rex* subsp. *rex**;* Table S4. Genetic diversity and differentiation parameters for the combined cpDNA in 11 populations of *R. rex* subsp*. rex**;* Table S5. Parameters of neutrality tests based on cpDNA of *R. rex* subsp. *rex.*
