# Supplementary material for: Genetic Diversity and Population Structure of Rhododendron rex Subsp. rex Inferred from Microsatellite Markers and Chloroplast DNA Sequences
Source: Plants (Basel). 2020 Mar 7;9(3):338. doi: 10.3390/plants9030338 (PMC7154904; doi:10.3390/plants9030338)
Supplement: Supplementary file 1 [file plants-09-00338-s001.zip › plants-727144-SI/Supplementary File/Supplementary Table 1.pdf]

Supplementary Material

Genetic diversity and population structure of *Rhododendron rex* subsp. *rex* inferred from microsatellite makers and chloroplast DNA sequences

Authors: Xue Zhang, Yuan-Huan Liu, Yue-Hua Wang, Shi-Kang Shen*

School of Life Sciences, Yunnan University, Kunming No. 2 Green lake North road Kunming, Yunnan, 650091, China.

***Correspondence author:** Shi-Kang Shen (ssk168@ynu.edu.cn; yunda123456@126.com)

Supplementary Table 1 The information of 14 microsatellite primers for the *R. rex* subsp. *rex*

Primers	Repeat types	Primers sequence	Tm (°C)
R-21	(AC)3C(CA)6	F: GCAACCTACATTCTCAACAT<br>R: ACTACAAGCCTGAACCACAT	56
R-25	(AC) 13	F: GATGATAAGCATGAACGTGGC<br>R: CGGTCTTGATTGTGGTGAT	56
R-27	(AG) 2 GA (AG) 5	F: CAAAATCCACCAAGAACGAC<br>R: ACAAATCTTCCATCTCACCC	54
R-30	(CT) 14	F: AACCTCCTCAAATCGACAAC<br>R: GAAGCAAACAAATATCCCAC	54
R-31	(AG) 14	F: CAAAAGTTTTTCAGCGGGTA<br>R: AAGAAACAGCCGTATGCGAT	54
R-32	(CT) 13	F: TTCACCTCCTGCGGCACAA<br>R: CGAGAGCATCGGCGTAGTAT	54
R-40	(GA)9	F: AAGGTGATCGTGTCGGAATA<br>R: TGCCTCTAACTACTTGCTCC	56
R-42	(AG)10	F: GTTGTAGATGCCGAGGAG<br>R: AACCACCAAAGCAGACCC	62
R-49	(GA)8	F: AAGGTGATCGTGTCGGAATA<br>R: TGCCTCTAACTACTTGCTCC	56
R-56	(AGG)3(AG)9	F: AGATCCGTATTTCTTGAGG<br>R: CAAAACCCACTTGTTAGAT	56
