# Supplementary material for: Genetic Diversity and Population Structure of Rhododendron rex Subsp. rex Inferred from Microsatellite Markers and Chloroplast DNA Sequences
Source: Plants (Basel). 2020 Mar 7;9(3):338. doi: 10.3390/plants9030338 (PMC7154904; doi:10.3390/plants9030338)
Supplement: Supplementary file 1 [file plants-09-00338-s001.zip › plants-727144-SI/Supplementary File/Supplementary Table 2.pdf]

Supplementary Material

Genetic diversity and population structure of *Rhododendron rex* subsp. *rex* inferred from microsatellite makers and chloroplast DNA sequences

Authors: Xue Zhang, Yuan-Huan Liu, Yue-Hua Wang, Shi-Kang Shen*

School of Life Sciences, Yunnan University, Kunming No. 2 Green lake North road Kunming, Yunnan, 650091, China.

***Correspondence author:** Shi-Kang Shen (ssk168@ynu.edu.cn; yunda123456@126.com)

Supplementary Table 2 Summary of the 10 microsatellite loci used to the 11 populations of *Rhododendron rex* subsp. *rex*

Locus	*Ra*	N_A	A_E	*I*	*Ho*	H_E	*UHE*	F_{IS}	F_{IT}	F_{ST}	H_S	H_T	G_{ST}	G'_{ST}	*Dest*
R-21	7.618	26	10.364	1.984	0.720	0.822	0.847	0.124	0.196	0.082	0.852	0.899	0.052	0.057	0.047
R-25	4.016	14	5.091	1.002	0.506	0.504	0.518	-0.005	0.228	0.232	0.520	0.657	0.209	0.225	0.137
R-27	2.312	10	2.636	0.423	0.034	0.220	0.228	0.846	0.869	0.145	0.233	0.258	0.099	0.108	0.026
R-30	8.210	30	10.727	1.999	0.814	0.811	0.836	-0.003	0.119	0.122	0.837	0.926	0.096	0.105	0.089
R-31	4.548	14	4.455	0.857	0.368	0.422	0.433	0.128	0.430	0.346	0.437	0.646	0.324	0.345	0.209
R-32	6.819	19	7.182	1.532	0.575	0.670	0.722	0.178	0.326	0.179	0.727	0.855	0.149	0.162	0.128
R-40	2.815	8	3.364	0.736	0.286	0.416	0.428	0.313	0.493	0.262	0.433	0.565	0.233	0.25	0.131
R-42	3.642	14	3.455	0.586	0.311	0.300	0.309	-0.038	0.452	0.471	0.309	0.569	0.456	0.48	0.259
R-49	3.269	8	4.000	0.880	0.327	0.472	0.486	0.308	0.460	0.219	0.492	0.606	0.188	0.203	0.114
R-56	6.484	26	7.455	1.592	0.703	0.730	0.750	0.038	0.190	0.158	0.754	0.869	0.132	0.143	0.115
Total		169													
