# Supplementary material for: Genetic Diversity and Population Structure of Rhododendron rex Subsp. rex Inferred from Microsatellite Markers and Chloroplast DNA Sequences
Source: Plants (Basel). 2020 Mar 7;9(3):338. doi: 10.3390/plants9030338 (PMC7154904; doi:10.3390/plants9030338)
Supplement: Supplementary file 1 [file plants-09-00338-s001.zip › plants-727144-SI/Supplementary File/Supplementary Table 3.pdf]

***Supplementary Material***

**Genetic diversity and population structure of *Rhododendron rex* subsp. *rex* inferred from microsatellite makers and chloroplast DNA sequences**

**Authors:** Xue Zhang, Yuan-Huan Liu, Yue-Hua Wang, Shi-Kang Shen*

School of Life Sciences, Yunnan University, Kunming No. 2 Green lake North road Kunming, Yunnan, 650091, China.

***Correspondence author:** Shi-Kang Shen (ssk168@ynu.edu.cn; yunda123456@126.com)

Supplementary Table 3 *P*-value of Hardy-Weinberg equilibrium test for 11 populations of *R. rex* subsp. *rex*

| Population | R-21 | R-25 | R-27 | R-30 | R-31 | R-32 | R-40 | R-42 | R-49 | R-56 | All loci |
|---|---|---|---|---|---|---|---|---|---|---|---|
| BJS | 0.065 ns | 0.971 ns | 0.014 * | 0.907 ns | - | 0.355 ns | 0.014 * | 0.624ns | 0.014 * | 0.971 ns | 0.998ns |
| DLT | 0.006 ** | 0.007 ** | 0.000 *** | 0.218 ns | 0.110 ns | 0.140 ns | 0.286 ns | - | 0.503 ns | 0.027 * | 0.998ns |
| QLB1 | 0.447 ns | 0.000 *** | 0.913 ns | 0.088 ns | 0.046 * | 0.819 ns | 0.001 *** | 0.997ns | 0.014 * | 0.080 ns | 0.999ns |
| QLB2 | 0.000 *** | 0.000 *** | 0.000 *** | 0.809 ns | 0.952 ns | 0.320 ns | 0.011 * | 0.976ns | 0.004 ** | 0.254 ns | 1.000ns |
| QLB3 | 0.541 ns | 0.353 ns | - | 0.351 ns | 0.667 ns | 0.935 ns | 0.009 ** | 0.724ns | 0.006 ** | 0.551 ns | 0.975ns |
| GDX | 0.095 ns | 0.002 ** | 0.000 *** | 0.142 ns | 0.000 *** | 0.857 ns | 0.525 ns | 0.908ns | 0.618 ns | 0.979 ns | 1.000ns |
| BCL | 0.002 ** | 0.008 ** | 0.000 *** | 0.359 ns | 0.911 ns | 0.007 ** | 0.989 ns | 0.900ns | 0.972 ns | 0.000 *** | 1.000ns |
| LJS | 0.000 *** | 1.000 ns | 0.000 *** | 0.853 ns | 0.898 ns | 0.000 *** | 0.121 ns | 1.000ns | 0.327 ns | 0.757 ns | 1.000ns |
| JZS | 0.468 ns | 0.535 ns | 0.000 *** | 0.996 ns | 0.000 *** | 0.109 ns | 0.000 *** | 0.513ns | 0.012 * | 0.123 ns | 1.000ns |
| LZS | 0.723 ns | 0.090 ns | - | 0.978 ns | 0.868 ns | 0.285 ns | 0.901 ns | 0.248ns | 0.020 * | 0.307 ns | 0.976ns |
| YS | 0.632 ns | 0.990 ns | 0.000 *** | 0.000 *** | 0.060 ns | 0.933 ns | 0.394 ns | 0.494ns | 0.526 ns | 0.328 ns | 0.999ns |

| | | | | | | | | | | |
|---|---|---|---|---|---|---|---|---|---|---|
| All pop. | 1.000 ns | 0.990 ns | 1.000 ns | 0.971 ns | 1.000 ns | 1.000 ns | 1.000 ns | 0.571ns | 1.000 ns | 0.997 ns | 1.000ns |

Note：-，Monomorphic；ns，non-significance；*，$P$＜0.05，significant difference；**，most significant difference；***，P＜0.001，most significant difference.
