# Supplementary material for: Genetic Diversity and Population Structure of Rhododendron rex Subsp. rex Inferred from Microsatellite Markers and Chloroplast DNA Sequences
Source: Plants (Basel). 2020 Mar 7;9(3):338. doi: 10.3390/plants9030338 (PMC7154904; doi:10.3390/plants9030338)
Supplement: Supplementary file 1 [file plants-09-00338-s001.zip › plants-727144-SI/Supplementary File/Supplementary Table 4.pdf]

Supplementary Material

Genetic diversity and population structure of *Rhododendron rex* subsp. *rex* inferred from microsatellite makers and chloroplast DNA sequences

Authors: Xue Zhang, Yuan-Huan Liu, Yue-Hua Wang, Shi-Kang Shen*

School of Life Sciences, Yunnan University, Kunming No. 2 Green lake North road Kunming, Yunnan, 650091, China.

***Correspondence author:** Shi-Kang Shen (ssk168@ynu.edu.cn; yunda123456@126.com)

Supplementary Table 4 Genetic diversity, differentiation parameters for the combined cpDNA in 11 populations of *R. rex* subsp. *rex*

Primer	H_S	H_T	*Gst*	*Nst*
cpDNA	0.243	0.522	0.534	0.728

Note: H_S=the mean heterozygosity within population; H_T=total genetic diversity for species.
