# Supplementary material for: Genetic Diversity and Population Structure of Rhododendron rex Subsp. rex Inferred from Microsatellite Markers and Chloroplast DNA Sequences
Source: Plants (Basel). 2020 Mar 7;9(3):338. doi: 10.3390/plants9030338 (PMC7154904; doi:10.3390/plants9030338)
Supplement: Supplementary file 1 [file plants-09-00338-s001.zip › plants-727144-SI/Supplementary File/Supplementary Table 5.pdf]

Supplementary Material

Genetic diversity and population structure of *Rhododendron rex* subsp. *rex* inferred from microsatellite makers and chloroplast DNA sequences

Authors: Xue Zhang, Yuan-Huan Liu, Yue-Hua Wang, Shi-Kang Shen*

School of Life Sciences, Yunnan University, Kunming No. 2 Green lake North road Kunming, Yunnan, 650091, China.

***Correspondence author:** Shi-Kang Shen (ssk168@ynu.edu.cn; yunda123456@126.com)

Supplementary Table 5 Parameters of neutrality tests based on cpDNA of *R. rex* subsp. *rex*

Primers	Tajima's *D*	Fu and Li's *D**	Fu and Li's *F**	Fu's *Fs*	SSD	HRag
cpDNA	0.496	1.615*	1.427	2.285	0.069	0.216
